# Bilateral Defect Cutting Strategy for Sawn Timber Based on Artificial Intelligence Defect Detection Model

**DOI:** 10.3390/s24206697

**Published:** 2024-10-18

**Authors:** Chenlong Fan, Zilong Zhuang, Ying Liu, Yutu Yang, Haiyan Zhou, Xu Wang

**Affiliations:** College of Mechanical and Electronic Engineering, Nanjing Forestry University, Nanjing 210037, China; fancl@njfu.edu.cn (C.F.); zzl0702@njfu.edu.cn (Z.Z.); yangyutu@njfu.edu.cn (Y.Y.); zhouhaiyanzj@njfu.edu.cn (H.Z.); schuewang@njfu.edu.cn (X.W.)

**Keywords:** artificial intelligence, timber processing, bilateral sawing, defect detection

## Abstract

Solid wood is renowned as a superior material for construction and furniture applications. However, characteristics such as dead knots, live knots, piths, and cracks are easily formed during timber’s growth and processing stages. These features and defects significantly undermine the mechanical characteristics of sawn timber, rendering it unsuitable for specific applications. This study introduces BDCS-YOLO (Bilateral Defect Cutting Strategy based on You Only Look Once), an artificial intelligence bilateral sawing strategy to advance the automation of timber processing. Grounded on a dual-sided image acquisition platform, BDCS-YOLO achieves a commendable mean average feature detection precision of 0.94 when evaluated on a meticulously curated dataset comprising 450 images. Furthermore, a dual-side processing optimization module is deployed to enhance the accuracy of defect detection bounding boxes and establish refined processing coordinates. This innovative approach yields a notable 12.3% increase in the volume yield of sawn timber compared to present production, signifying a substantial leap toward efficiently utilizing solid wood resources in the lumber processing industry.

## 1. Introduction

China, as a significant consumer of wood and a major exporter of wood products, currently relies on substantial wood imports. Export volumes significantly lag behind import quantities, resulting in a substantial trade deficit. Additionally, China grapples with several challenges within the domain of defect-oriented processing of solid wood materials [1]. Many small-scale wood processing facilities exhibit relatively old processing methods, often relying on manual marking and sorting, leading to notable limitations [2]. Developed countries have established relatively comprehensive solid wood processing systems, which provide significant advantages in the depth and breadth of wood processing. In the sawmilling process, the acceptable types, positions, sizes, and quantities of features vary among woods destined for different purposes [3]. Consequently, relevant enterprises have established diverse standards to categorize and grade round and sawn timber, significantly impacting the pricing of logs and sawn timber. Therefore, the automatic recognition, classification, and grading of timber defects constitute a pivotal prerequisite for enhancing both timber utilization and the value of wood products.

With the advancement of detection technologies, wood inspection methods exhibit characteristics of multiscale capabilities [4]. These methods can assess various properties of wood, such as density [5], elastic modulus, and tensile and compressive strengths, while also allowing for the observation of its microscopic features, including wood fibers and cellular conduits. Moreover, they can scrutinize surface attributes such as color and texture and internal defects within the wood. In the past 20 years, the primary means of inspecting lumbers encompass X-ray [6], ultrasonic [7], microwave radiation [8], visual [9], and acoustic emission techniques [10]. Subsequent to acquiring data regarding timber, conventional inspection methods involve the utilization of manually designed feature extraction algorithms. These encompass procedures such as the one-dimensional wavelet transform for time series signals, local binary patterns (LBP) for two-dimensional images, and grayscale co-occurrence matrices, followed by further analysis based on the extracted features [11]. These detection methods serve as valuable tools for the further processing of solid wood, establishing themselves as the cornerstone for the preferred selection and processing of solid wood materials. Among all the detection methods, the image-based inspection methods for solid-sawn timber demonstrate superior expressiveness in detecting defects. They visually represent defect location, size, and type [12,13].

Consequently, the utilization of image-based techniques for wood defect detection has become a focal point of current research. Principal component analysis was conducted on the spectral information of four types of defects (dead knots, live knots, pinholes, and cracks) collected from larch wood using a near-infrared spectrometer, and the data were used as inputs for a backpropagation neural network, achieving a classification accuracy of over 92.0% for defects [14]. Similarly, LBP features and a histogram of the oriented gradient from wood images were fused. After dimensionality reduction through principal component analysis, they employed support vector machines to classify knots, live knots, insect damage, cracks, damage, wood structural defects, and wood processing defects, achieving a maximum classification accuracy of 98.9% [15]. These feature extraction methods yield satisfactory results in controlled laboratory settings. However, they exhibit limited robustness, rendering them susceptible to disruptions from external factors. Within the intricate landscape of industrial environments, achieving and maintaining high detection accuracy over prolonged periods proves challenging. Conversely, integrating deep learning techniques with conventional detection methodologies and extensive modeling of defect data bolsters the resilience of wood defect detection systems to external interference. This synergy empowers the rapid and precise identification of wood defects.

Convolutional neural networks have long been at the forefront of deep learning and have found extensive utility in surface defect detection. CNN-based deep learning technology has demonstrated its capability in terms of image classification because it can automatically detect and extract high-level image features from labelled image data [16,17]. In image classification, a combination of transfer learning and a ResNet-34 convolutional neural network was proposed for knot recognition in wood [18]. The experimental outcomes revealed a notable enhancement in the overall classification accuracy of knots, attaining a level of 98.69%, surpassing the performance of GoogleNet, AlexNet, and VGGNet-16. In semantic segmentation, the Faster R-CNN framework was adopted to identify surface defects on wood veneers [19]. Employing four distinct pre-trained models (AlexNet, VGG16, BN-Inception, and ResNet152) for transfer learning, the experimental results indicate that the ResNet152 pre-trained model achieved the highest average recognition accuracy, reaching 80.6%. These models are predominantly employed for characterizing the types of wood defects within images, yet they do not delineate their spatial coordinates. As a prominent focal point in contemporary deep learning for object detection, YOLO swiftly accomplishes target detection [20]. It accurately conveys detected defect classification and spatial positioning, aligning more closely with real-world production applications. A deep learning defect detection and recognition framework based on YOLOv4 was constructed to effectively ascertain the location and types of four defects (live knots, dead knots, cracks, and insect holes) on the surface of domestically produced spruce structural timber, achieving an average recognition rate of 96.7%. Leveraging the YOLOv5 algorithm model [21,22], surface knot defects in sawn timber were identified. Compared to YOLOv3 SPP and Faster R-CNN, the YOLOv5 model exhibits a notably heightened recognition accuracy.

In the current landscape, where wood detection methods are becoming increasingly diverse and mature, research focuses on utilizing robust and effective data mining algorithms to process detection data. This process aims to guide further processing strategies effectively. Methods for recognizing and classifying wood features based on deep learning offer the advantage of directly taking images as inputs. This obviates the need for labor-intensive and intricate pre-image feature extraction, sparing one from manually designing visual features. However, it is noteworthy that the prevailing detection algorithms are predominantly tailored for laboratory settings. These algorithms exhibit considerable disparity from practical application scenarios, posing a noteworthy discrepancy between laboratory settings and industry settings.

In summary, the defects of solid wood in practical applications greatly hinder its engineering application. Defects should be removed during processing according to production requirements. Detecting defect types and positions on solid wood panels is essential for subsequent intelligent processing. Therefore, an intelligent calculation method of machining coordinates based on the double-sided defect detection of solid wood panels is proposed in this manuscript. A dataset consisting of four types of defects—dead knots, live knots, cracks, and pith—was constructed. Then, defect detection models were built using the YOLOv7, YOLOX, and YOLOv5 algorithms, and their performance was compared. Based on the optimal defect detection model, the location and classification of double-sided defects of solid wood panels were realized, which lays a technical foundation for the automatic and accurate removal of defects in the future.

## 2. Materials and Methods

### 2.1. Image Acquisition

**Figure 1:**
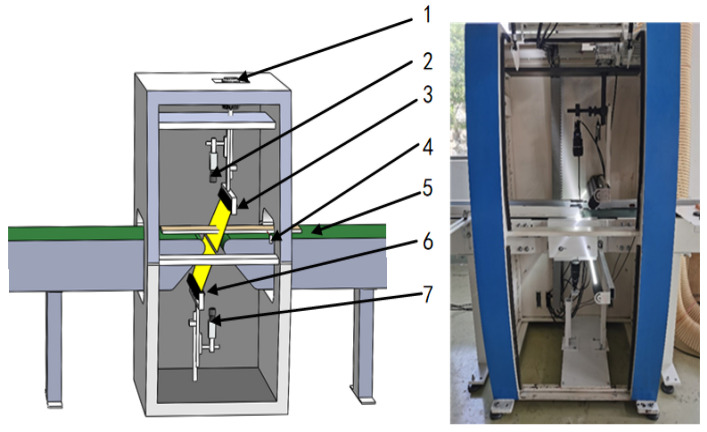
Image acquisition platform 1. motor adjustment; 2. top camera; 3. top illumination; 4. photoelectric switch; 5. conveyor belt; 6. bottom illumination; and 7. bottom camera.

The self-developed image acquisition platform (Figure 1) involves the adjustment of the angles of the top and bottom linear light sources (AST-Vision, LCOL-300-25, Shanghai, China) to ensure the optical illumination of the sawn timber’s surface. Subsequently, the heights of the top and bottom cameras (DALSA, LA-GC-02K05B, Waterloo, ON, Canada) are fine-tuned to ensure the clarity of the imaging of the sawn timber’s surface. Upon initiating the equipment, the solid-sawn timber advances on the conveyor belt (Hengshuntong, HST-01, Shenzhen, China). It passes over the photodetector switches on the image acquisition platform in Figure 1, triggering the image capture by the top and bottom cameras. These cameras capture the original images of the sawn timber’s surface, with an image size of 2048 × 17,000 pixels.

The primary hardware components employed in the image acquisition platform are summarized in Table 1.

### 2.2. Image Preprocessing

**Figure 2:**
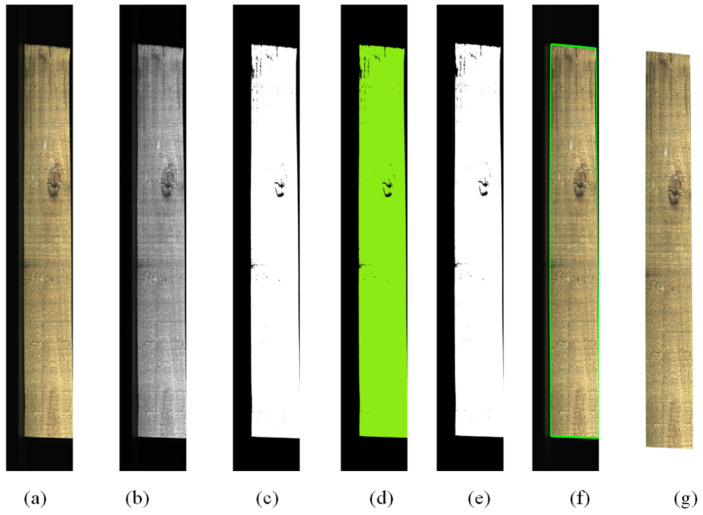
Image preprocessing: (**a**) original image; (**b**) grayscale; (**c**); image binarization (**d**); connected component analysis (**e**); ROI selection (**f**); erosion and filling; and (**g**) image cutting.

Due to the inherent variation in the dimensions of sawn timber, the acquired images often contain background elements. Furthermore, the significant disparity between the sawn timber and the conveyor belt necessitates the application of conventional image preprocessing techniques to eliminate the background from the images of sawn timbers. This process yields favorable results and is illustrated in Figure 2. The acquired image is initially converted into a grayscale representation, as shown in Figure 2b. Subsequently, the image undergoes threshold segmentation to transform it into a binary format, as shown in Figure 2c. The connectivity domain computation is then employed on the binary image, as shown in Figure 2d, to extract the Region-of-Interest (ROI) by isolating the largest connected domain, as shown in Figure 2e. Using the erosion morphological operation, fine serrations at the edges are eliminated, and small holes within the ROI are filled to generate the mask for the timber region, as shown in Figure 2f. Ultimately, guided by the mask region, the image of the timber is precisely cut out, as shown in Figure 2g.

By employing this method, the interference caused by the conveyor belt background is substantially mitigated. This streamlines the defect detection task in the sawn timber, allowing the focus to remain primarily on the timber pieces themselves. Consequently, the detection process narrows its scope to identifying defects on the solid-sawn timber as the target, treating the undamaged sections of the timbers as background (negative samples). This approach leads to superior detection performance.

### 2.3. Dataset Setup

The sawn timber used in the test was sourced from the building decoration materials market and was dry, finished sawn timber with a size of 1200 mm × 150 mm × 20 mm, and it consisted of five species: Cerasus pseudocerasus, Fagus longipetiolata Seem, Pinus yunnanensis Franch, Fraxinus mandschurica Rupr, and Betula platyphylla Suk. The color and texture variations among these five tree species are considerable. Pseudocerasus sawn timber is more towards dark red, while Fraxinus mandschurica Rupr sawn timber is more towards white. These differences helped to ensure diversity within the dataset samples to a certain extent. Given that most timber undergoes an initial screening during production and distribution to downstream markets, the prevalence of flawed sawn timber in our purchases was notably low. Two hundred images of sawn timber were selected for input into the image acquisition platform, as shown in Figure 1. From these, a dataset of 400 images of sawn timber was assembled.

**Figure 3:**
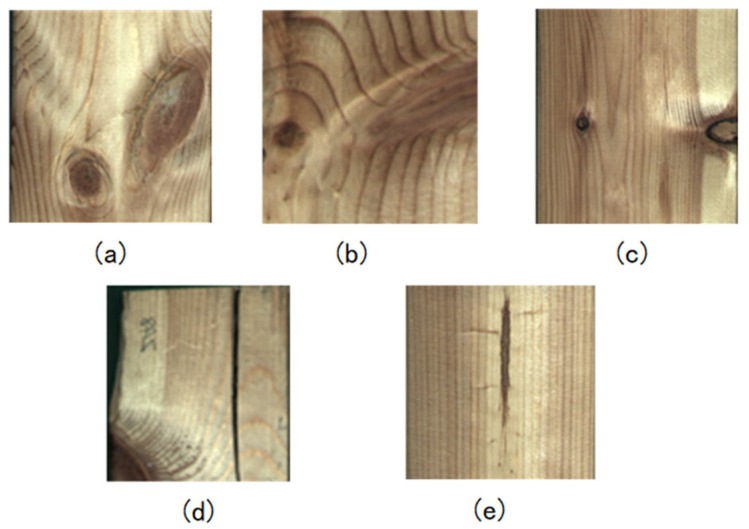
Defects in dataset (**a**) live knot (V); (**b**) live knot (P); (**c**) dead knot; (**d**) crackle; and (**e**) pith.

In light of the diverse array of defects associated with sawn timber and the severe imbalance in their distribution, the production process predominantly centers on the identification of knot defects (live knots and dead knots) and cracks, as shown in Figure 3. Live knots originate from the live branches of trees, exhibiting dense growth rings tightly interwoven with the surrounding wood. Figure 3a showcases the appearance resulting from cutting in the direction perpendicular to the branch, while Figure 3b shows the shape following cutting parallel to the branch direction.

On the other hand, dead knots originate from the dead branches of trees, and their growth rings are detached from or partially disengaged from the surrounding wood, as shown in Figure 3c. In the state of the original log, due to the separation of wood along the grain direction, cracks develop, resulting in fissures on the surface of the log and the board, as exemplified in Figure 3d. These cracks predominantly emerge during the preservation of the wood. In this batch of sawn timber, obtained through the tangential cutting method and possessing a cutting location proximate to the pith, many species exhibited extensive, slender, dark stripes, as depicted in Figure 3e. These stripes, akin to knots and cracks, introduce significant differences in mechanical properties compared to the surrounding wood, affecting the use of the sawn timber.

The original images had a pixel resolution of 2048 × 17,000, with only a tiny portion of the images containing defects. Utilizing convolutional neural networks on such vast images significantly escalates computational demands and consumes excessive memory, substantially impeding the network’s operational speed. Therefore, by randomly cropping the images and selecting the samples with defects, images containing wood surface defects were extracted from the original images for network training.

Following processing, the acquired original images were partitioned into images of 200 × 200 pixels, encompassing various defects. This procedure resulted in an initial dataset of approximately 1500 images. Supervised learning requires sample annotations to train the target detection model. This entails iteratively updating the parameters of the constructed neural network on the training set and subsequently validating the algorithm accuracy on the test set.

**Figure 4:**
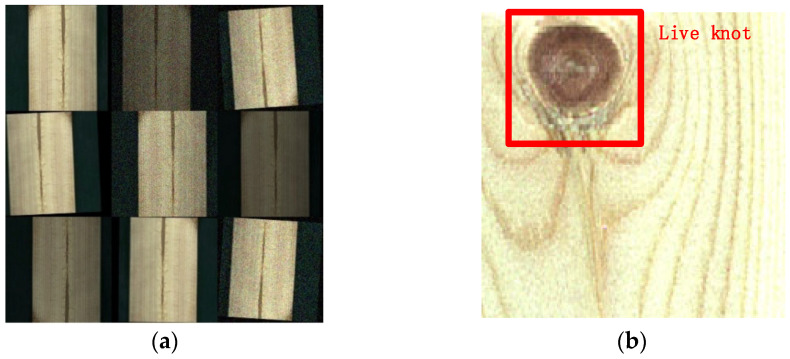
Dataset producing (**a**) data augmentation; (**b**) defect annotation.

Consequently, 1050 images of the original dataset were randomly selected as the training set, with the remaining 450 images reserved for testing. To enhance training efficacy, three data augmentation techniques were applied (Figure 4), expanding the original training set by a factor of 10: (1) mirroring the images along their horizontal axis, (2) introducing random uniform noise, and (3) applying random rotations by certain angles. The training set was expanded to 10,500 images. The LabelImg software (2023.1.3 Professional Edition, JetBrains, Prague, Czech Republic)was used to annotate four types of defects in the preprocessed images of sawn timber; defect types and their distribution are shown in Table 2.

### 2.4. Bilateral Sawing Coordinate Transformation

**Figure 5:**
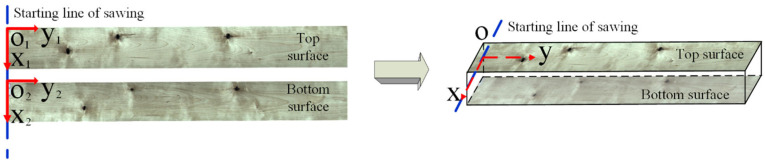
Bilateral sawing coordinate transformation.

Synchronizing image acquisition between the top and bottom cameras proved challenging, prompting the utilization of the preprocessing workflow depicted in Figure 2. This workflow serves to extract the sawn timber images. The images are aligned with an established coordinate system, wherein one side of the sawn timber entering the image acquisition platform forms the *x*-axis, and the internal corner serves as the origin of the coordinate system. The *y*-axis aligns with the direction of entry. When disregarding thickness, this coordinate system’s construction method facilitates the alignment of the image coordinates of the top and bottom surfaces of the sawn timber, as shown in Figure 5.

Simultaneously, as the sawn timber enters the sawing device, its origin coincides with the image coordinates. The transformation of defect coordinates on the image of the timber to the actual processing coordinates requires careful consideration. Maintaining a consistently stable speed with a conveyor controlled by a stepper motor in the image acquisition setup is challenging. Inconsistencies in speed can result in distortion and aberration in the images captured at a constant frame rate. Therefore, using encoders is crucial to synchronize the camera, enabling the mapping between the image coordinate system and the length of the wood panel. The specific derivation of this transformation process is outlined below.

The actual length of the sawn timber, denoted as $l_{real}$, is defined as the length of the sawn timber passing through the camera scanning area within a certain period, i.e., the integral of the sawn timber’s motion speed during the capture time, as shown in Equation (1). In the absence of relative motion with the conveyor belt, the speed of the sawn timber is equivalent to the speed of the conveyor belt.

(1) $$l_{real}=\int v\left(t\right)dt=\int k_{1}\omega \left(t\right)dt$$

where $v\left(t\right)$ represents the time-dependent conveyor belt speed, $k_{1}$ stands for the transmission coefficient between the drive motor and the conveyor belt, and $\omega \left(t\right)$ denotes the rotational speed of the drive motor. When the conveyor belt is operating normally, $k_{1}$ remains constant, allowing us to express it as shown in Equation (2).

(2) $$l_{real}=k_{1}\int \omega \left(t\right)dt$$

The length of the captured images of the sawn timber can be expressed as the integral of the product of the timber’s speed at the time of capture, i.e., the line frequency (how many lines are captured per minute) and the row frequency signal provided by the encoder of the drive motor, as shown in Equation (3).

(3) $$l_{image}=\int f\left(t\right)dt=\int k_{2}\omega \left(t\right)dt$$

where $f\left(t\right)$ represents the time-dependent camera capture speed, $k_{2}$ denotes the prescaling factor set when transmitting the encoder signal to the camera, and $\omega \left(t\right)$ stands for the rotational speed of the drive motor. As $k_{2}$ remains constant, it can be expressed as shown in Equation (4).

(4) $$l_{image}=k_{2}\int \omega \left(t\right)dt$$

From Equations (3) and (4), there is Equation (5).

(5) $$l_{real}=(k_{1}/k_{2})l_{image}=Kl_{image}$$

Therefore, the actual length of the timber is in direct proportion to the image length. By extracting the sawn timber area and measuring the actual length compared to the image length, we can obtain the scaling factor K to convert the image length to the actual length.

### 2.5. Image-Operation-Based Bilateral Sawing Coordinate Optimization Module

The positions and types of defects on sawn timber are random. Generating processing coordinates from detected defect coordinates involves considering the following three aspects:

(1) Merging features on both sides

**Figure 6:**
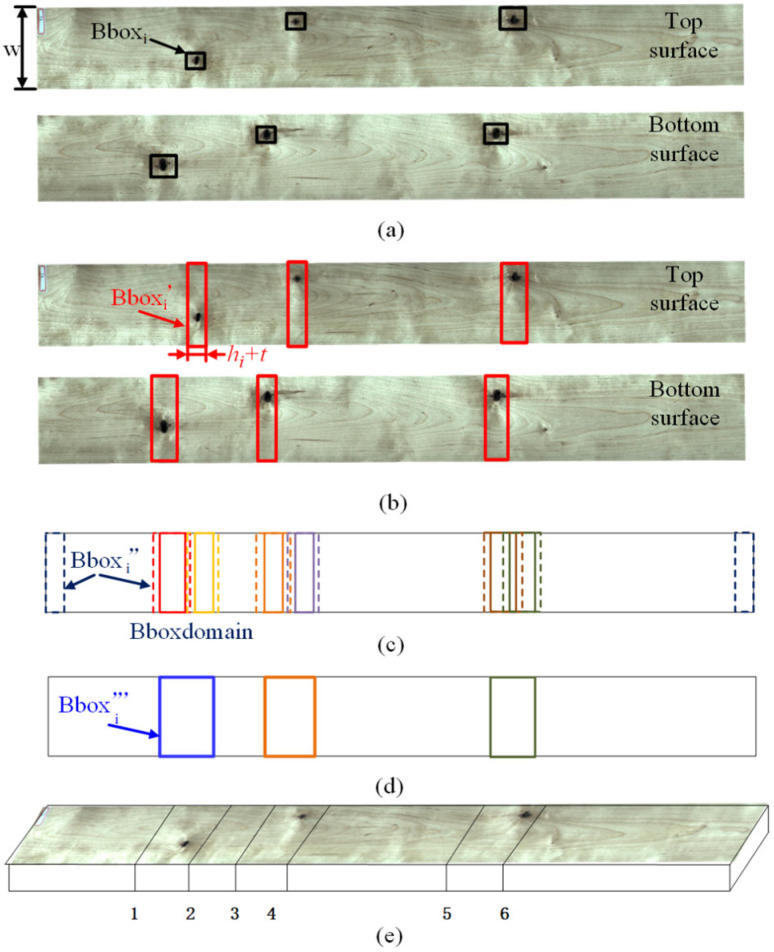
Bilateral sawing coordinate optimization flowchart: (**a**) double-sided defect detection; (**b**) detection box extension; (**c**) detection box dilatation; (**d**) detection box connectivity; and (**e**) optimized results.

In cases where there are features that span both sides of a sawn timber, it is crucial to merge these defects. For instance, as shown in Figure 6a, the defect detection algorithm may identify six separate features if a sawn timber has three continuous dead knots. Since these knots are not perfectly vertical to the sawn timber’s surface and their relative positions on the top and bottom surfaces may not align, an algorithm needs to be designed to merge these top and bottom surface defects.

Based on the assumption of the same sawn timber shape on both sides, the coordinates of the top and bottom surfaces are aligned to the XOY coordinate system by the starting line of sawing so that the detection bounding box on the top and bottom surfaces do not need to be changed numerically. Assuming that n defects are detected in total, the bounding boxes for defects on the top and bottom surfaces are denoted as $Bbox_{i}$, as shown in Equation (6).

(6) $$Bbox_{i}=\left(x_{i},y_{i},w_{i},h_{i}\right),i=1,2,\ldots n$$

where $x_{i}$ and $y_{i}$ represent the feature’s central coordinates and $w_{i}$ and $h_{i}$ denote the defect’s length and width.

(2) Considering saw blade thickness and eliminating short sawn timber

Saw blades have a certain thickness, and contact with defects like knots can lead to rapid blade wear. Therefore, it is essential to account for the thickness of the saw blade. Since the sawing of the wood only requires transverse truncation, the width of the original defect is not taken into account, and only the coordinates of the original defect in the length direction need to be considered. Instead, taking into account the sawblade thickness problem, it is necessary to widen the original defect detection bounding box at two sawblade thicknesses. Therefore, the saw blade thickness t is introduced, and the morphological operation method of expansion is used to expand the defect box to obtain $Bbo{x^{\prime }}_{i}$ in Equation (7), as illustrated in Figure 6b:

(7) $$Bbo{x^{\prime }}_{i}=\left(w/2,y_{i},w,h_{i}+t\right),i=1,2,\ldots n$$

Sawn timber that is too short has no practical value and will accumulate in the sawing area, affecting subsequent processing. Therefore, it is unnecessary to set processing points for such short pieces. So, a bounding box deformation method based on image operation is introduced.

All the widened rectangular boxes are collectively drawn on a rectangular shape of the same size as the sawn timber. Subsequently, an inflation operation is applied to all rectangles to simulate the minimum processing size a. Considering the offcuts during processing and the initial material, it is necessary to add a pre-material rectangle at both the beginning and end of the sawn timber. Therefore, all the boundary boxes can be represented as shown in Equation (8):

(8) $$Bbo{x^{\prime \prime }}_{i}=\left\{\begin{array}{c} \left(w/2,\left(t+a\right)/2,w,t+a\right),\;\;i=0\\ \left(w/2,y_{i},w,h_{i}+t+a\right),i=1,\;2,\;\ldots n\\ \left(w/2,l-\left(t+a\right)/2,w,t+a\right),\;\;i=n+1 \end{array}\right.$$

By this method, the candidate crop boxes are calculated, but these crop boxes need to be further merged to remove the redundant sawing lines. Specifically, if there is an overlap between two preselected boxes, then it means that the distance between the original two sawing lines is less than the thickness of the saw blade and the minimum permissible machining length, i.e., the distance between the two features is short, and thus the two defects need to be merged into a single process. If the two candidate boxes do not overlap, then the distance between the two defects is sufficiently large to make sense of the resulting sawn material after sawing, and therefore, the two defects need to be retained without merging.

(3) Image-operation-based sawing point optimization

As shown in Figure 6c, by connecting the component analysis on all the rectangles within $Bbox^{\prime \prime }$, a bounding rectangle of all the connected components Bboxdomain is obtained, as shown in Equation (9):

(9) $$Bboxdomain=connecteddomain(Bbox^{\prime \prime })$$

By considering the original rectangles $Bbo{x^{\prime }}_{i}$ within Bboxdomain as a whole, the largest bounding rectangle $Bbo{x^{\prime \prime \prime }}_{j}$ is obtained, as represented in Equation (10). All the regions on the lumber designated for processing are acquired, as depicted in Figure 6d:

(10) $$Bbo{x^{\prime \prime \prime }}_{j}=bundingrectangular\left(Bbox^{\prime }\right),Bbox^{\prime }\in Bboxdomain_{j}$$

As shown in Figure 6e, the starting and ending coordinates of all rectangles $Bbo{x^{\prime \prime \prime }}_{j}$ in the y-direction are used to determine the processing points. The calculation method for sawing point $y_{c}$ is presented in Equation (11):

(11) $$y_{c}=(K\times Bbo{x^{\prime \prime \prime }}_{j}\left(y_{j}-(h_{j}+t+a)/2\right),K\times Bbo{x^{\prime \prime \prime }}_{j}\left(y_{j}+(h_{j}+t+a)/2\right))$$

In case there are defects on the extracted section or its size falls below the minimum usable length, the entire section will be considered invalid for material yield and should be discarded. The formula for calculating the effective material yield is described in Equation (12):

(12) $$\eta =\sum l_{ik}/\sum l_{i}$$

where $l_{ik}$ represents the length of the k-th valid product piece on the i-th lumber, and $l_{i}$ is the length of the i-th lumber.

### 2.6. Defect Detection Module Based on YOLOv7

**Figure 7:**
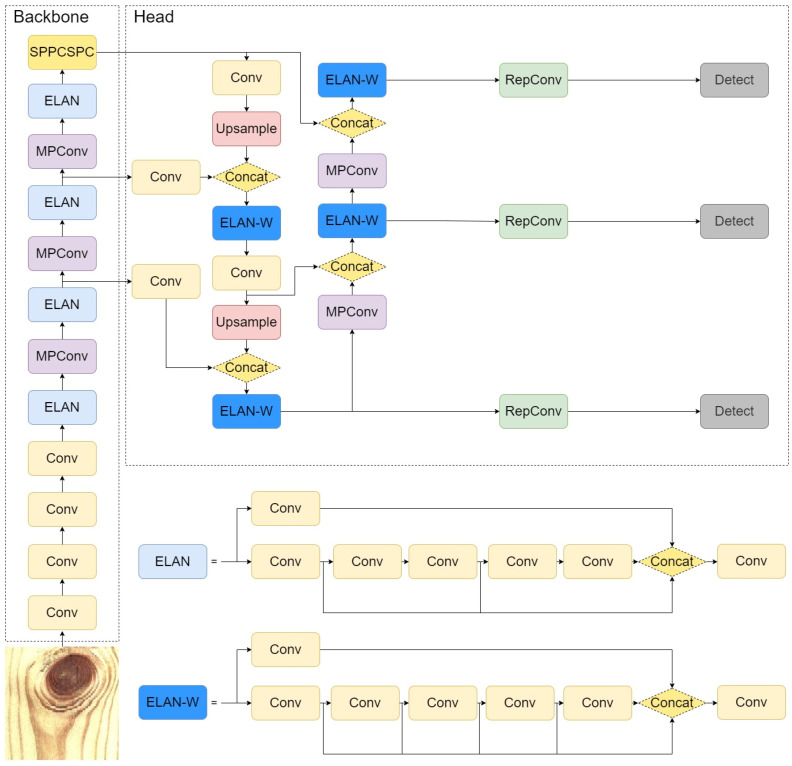
Defect detection model of wood panels based on YOLOv7.

In comparison to previous endeavors, YOLOv7 builds upon the foundation of YOLOv5 while assimilating a plethora of techniques developed by scholars in recent years for object detection tasks [23,24]. The structural framework of the bilateral timber sawing network based on YOLOv7 is depicted in Figure 7. In contrast to the earlier versions of YOLO, YOLOv7 has improved and optimized the YOLO framework by introducing novel deep learning network model techniques.

(1) Re-parameterized Convolution Module

The re-parameterized convolution module (RepConv) is a currently popular model architecture strategy. RepVGG, based on the VGG (Visual Geometry Group, a deep convolutional neural network architecture proposed by the Computer Vision Group at Oxford University) network design, is a multi-branch model that enhances performance through multiple branches during training [25]. In inference, it undergoes structural reparameterization to transform into a continuous straight-line VGG-style network with 3 × 3 convolutions and ReLU, accelerating inference speed. In convolutional neural network architectures, achieving inference acceleration using specific hardware can be incredibly intricate and burdensome. In contrast to additional customized hardware, the straight-line VGG-style network offers the advantages of simplicity and faster inference speed. However, the accuracy significantly diminishes when similar structures are directly applied to ResNet, DenseNet, or other network architectures. This is because the bypass route in RepConv disrupts the residual structure in ResNet and the inter-layer connections in DenseNet, reducing the gradient diversity among different feature maps. Therefore, removing the bypass route in the re-parameterized convolution structure mitigates its introduced accuracy decline.

(2) Efficient Long-range Attention Network Module

The efficient long-range attention network (ELAN) is an efficient network structure that, by controlling the shortest and longest gradient paths, enables the network to learn more features and enhances robustness. ELAN comprises two branches: one branch undergoes channel variation through a 1 × 1 convolution, and the other first encounters a 1 × 1 convolution module for channel adaptation. Subsequently, it passes through four 3 × 3 convolution modules for feature extraction.

Based on the YOLO backbone network, its input layer is changed to double-sided images, and the output layer is transformed to a bilateral sawing coordinate optimization module. The overall framework is shown in Figure 7.

### 2.7. Online Testing

**Figure 8:**
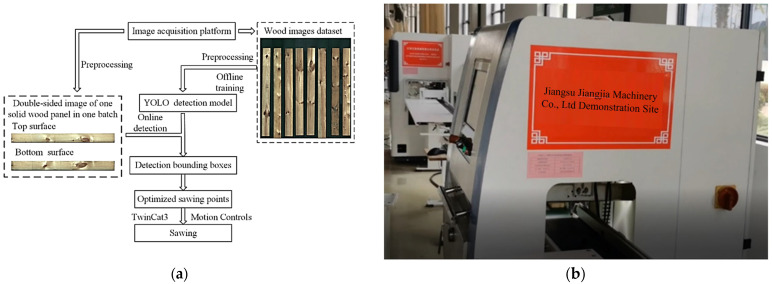
Online testing: (**a**) detection and processing workflow. (**b**) online testing device.

Equipment interconnection testing was carried out at Jiangsu Jiangjia Machinery Co., Ltd. (Yancheng, China), following the workflow depicted in Figure 8a. As shown in Figure 8b, the assembled equipment involved connecting the image acquisition system with the defect-oriented processing apparatus via a conveyor belt. Upon the solid-sawn lumbers being introduced through the loading port, the image acquisition apparatus captured images from both sides. Simultaneously, the upper-level computer computed the defect-oriented processing coordinates. Subsequently, the solid-sawn lumbers underwent processing within the defect-oriented processing apparatus, controlled by EtherCAT through the upper-level computer, resulting in the removal of defects from the solid-sawn lumbers.

## 3. Results

### 3.1. Defect Detection

The SGD optimizer was employed with an initial learning rate set at 0.01, and training was designated for 300 epochs. Training ceased when the loss function stabilized. The YOLOv5, YOLOX, and YOLOv7 defect detection networks were individually trained on the established dataset [26]. The training neural network was trained through the following computer hardware and software platforms, as shown in Table 3.

In this study, PR curves were used to evaluate the performance of the classification models. The P-R curve is the precision vs. recall curve. The more convex the curve is to the upper right corner, the better it is in the P-R curve. The P-R curve is obtained by obtaining different precision values, recalling at different thresholds, obtaining a series of points, plotting them in a P-R diagram, and connecting them sequentially to obtain the P-R curve. The test results in terms of the F1, recall, and precision metrics on the test set are shown in Table 4, with YOLOv7 having the highest mAP@0.5 of 0.94. Meanwhile, the reasoning time of YOLOv7, YOLOv5, and YOLOX algorithm models was 27 ms, 36 ms, and 35 ms, respectively. The YOLOv7 model showed excellent performance. The detection confusion matrix on the test set is presented in Figure 8.

**Figure 9:**
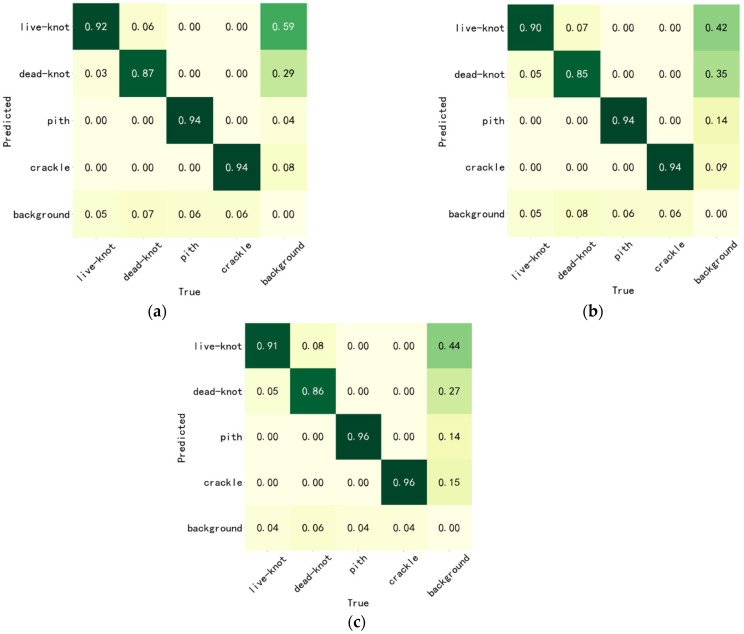
Confusion matrix in the test set. (**a**) Confusion matrix of YOLOv7 in the test set; (**b**) confusion matrix of YOLOv5 in the test set; and (**c**) confusion matrix of YOLOX in the test set.

Figure 9 shows that the recall rates for recognizing live knots, dead knots, piths, and cracks by the YOLOv7-based deep learning defect detection model were 0.92, 0.87, 0.94, and 0.94, respectively (Table 5). The YOLOv5-based deep learning defect detection model attained recall rates of 0.90, 0.85, 0.94, and 0.94 for the recognition of live knots, dead knots, pith, and cracks, while the YOLOX-based deep learning defect detection model achieved recall rates of 0.91, 0.86, 0.96, and 0.96 for the recognition of live knots, dead knots, pith, and cracks. All the defect detection models demonstrated the effective detection of piths and cracks, despite these two defects sharing the characteristic of elongated shapes; they yielded distinct outcomes with differing surrounding textures. Cracks result from mechanical damage and, as such, exert minimal influence on the surrounding textures [27,28,29]. Conversely, pith constitutes a structural defect arising during wood growth, with continuous texture variations in the elongated defect area and dark spots formed during growth. Notably, the YOLOX-based defect detection network model outperformed the others, highlighting the advantage of utilizing feature pyramids in detecting elongated defects.

However, all models exhibited limitations in the detection of live knots and dead knots [30]. Relatively, the recall rate for live knots surpassed that of dead knots, as dead knots were often missed or confused with live knots, leading to lower recognition rates. This challenge primarily arose from the difficulty of distinguishing the boundary between live knots and dead knots in certain situations, as well as some ambiguity in the dataset during its creation, ultimately affecting the subsequent defect detection models’ accuracy [31,32,33].

**Figure 10:**
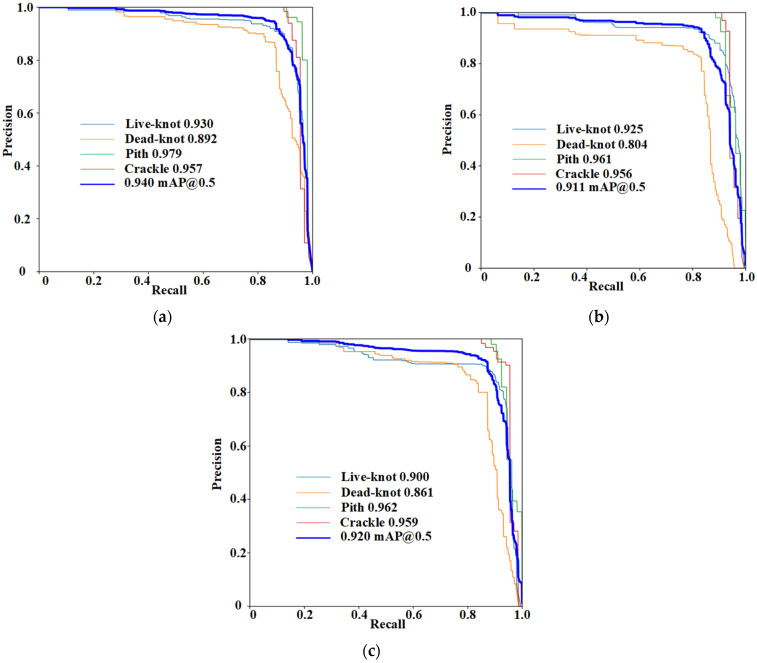
P-R curves in test set. (**a**) YOLOv7 P-R curves; (**b**) YOLOv5 P-R curves; and (**c**) YOLOX P-R curves.

Following Figure 10, the deep learning defect detection network model for sawn timber using YOLOv7 exhibited average precision rates of 0.93, 0.89, 0.98, and 0.96 for live knots, dead knots, pith detection, and crack recognition, respectively. The mean average precision stood at 0.94 (Table 6). In contrast, the deep learning defect detection network model for solid-sawn lumbers based on YOLOv5 achieved average precision rates of 0.93, 0.80, 0.96, and 0.96 for live knots, dead knots, pith detection, and crack recognition, resulting in a mean average precision of 0.91. Meanwhile, the solid-sawn lumber deep learning defect detection network model based on YOLOX demonstrated average precision rates of 0.90, 0.86, 0.96, and 0.96 for live knots, dead knots, pith detection, and crack recognition, with a mean average precision of 0.92.

In alignment with the findings from Figure 9, all the defect detection models exceled in identifying piths and cracks, while their performance in detecting dead knots and live knots lagged, thereby diminishing the overall detection efficacy. The YOLOv7-based deep learning object detection network model distinguished itself with a higher overall average accuracy, attesting to its commendable detection performance.

**Figure 11:**
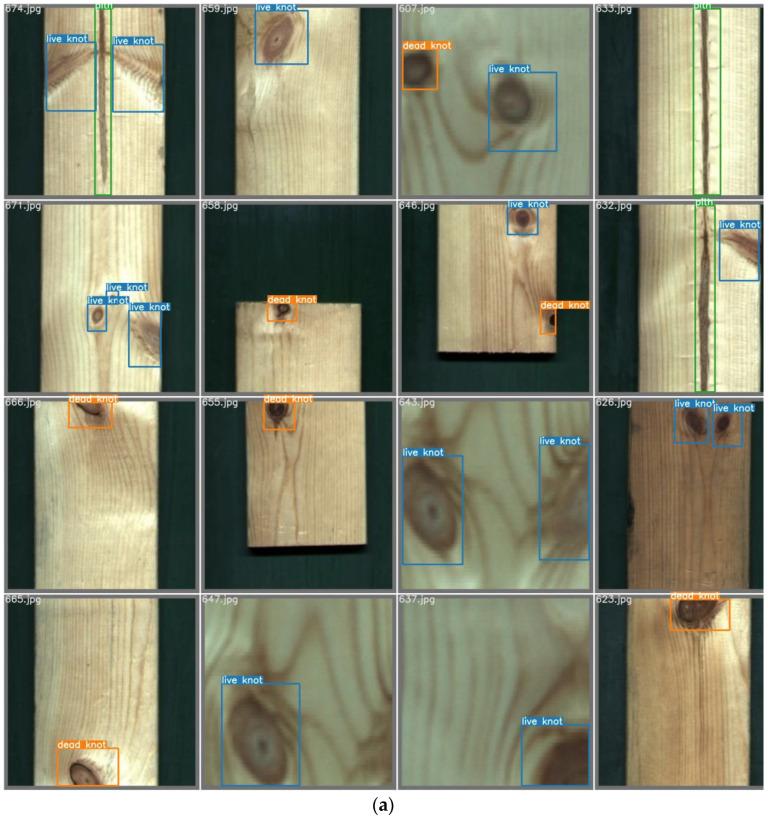

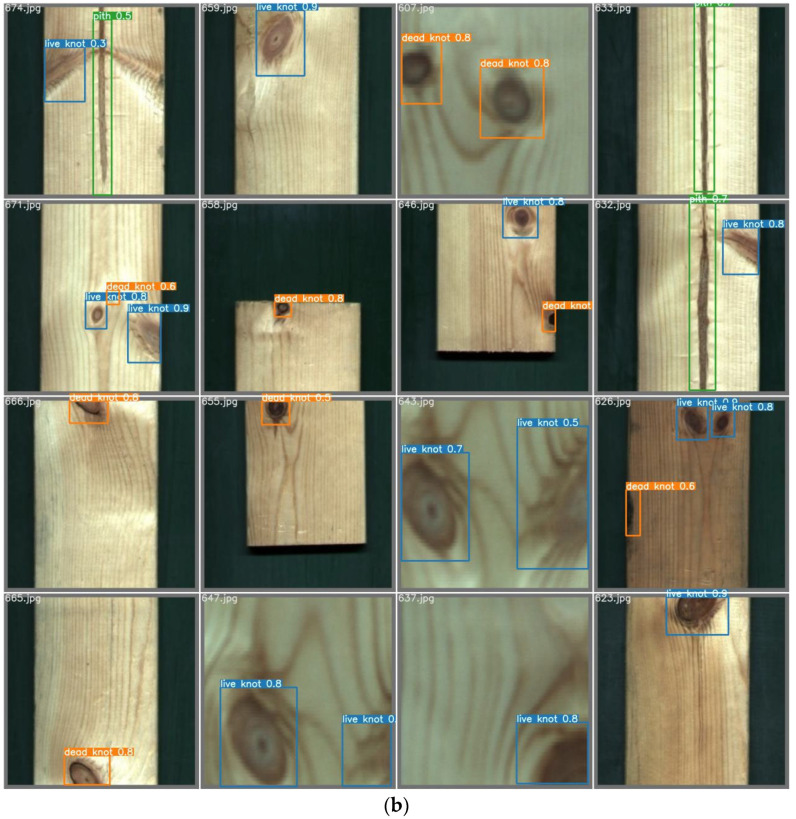
Detection result of the test set. (**a**) True value in the test set; and (**b**) prediction in the test set.

To evaluate the detection performance of the model, we selected anomalous test results from the defect detection model for solid-sawn lumbers based on YOLOv7. A subset of the detection results is illustrated in Figure 11. Figure 11a presents the ground truth annotations on the test set images, while Figure 11b displays the results from the solid-sawn lumber defect detection model based on YOLOv7.

In “674.jpg”, one live knot was missed due to its excessively light color. In “607.jpg”, a live knot was erroneously classified as a dead knot due to the dark color of its edges. In “671.jpg”, a live knot was misidentified as a dead knot because of its excessively silver appearance. In “626.jpg”, a shadow along the edge of the lumber was erroneously labeled as a dead knot. Lastly, in “647.jpg”, a section of the lumber with a dense texture was inaccurately classified as a live knot.

### 3.2. Online Sawing Test

The processing of 100 solid-sawn lumbers was carried out. Using a conventional fixed-length truncation approach, the yield amounted to 69.2%. In contrast, employing an intelligent processing scheme yielded a remarkable 81.5%, reflecting a notable enhancement of 12.3% ineffective material yield.

**Figure 12:**
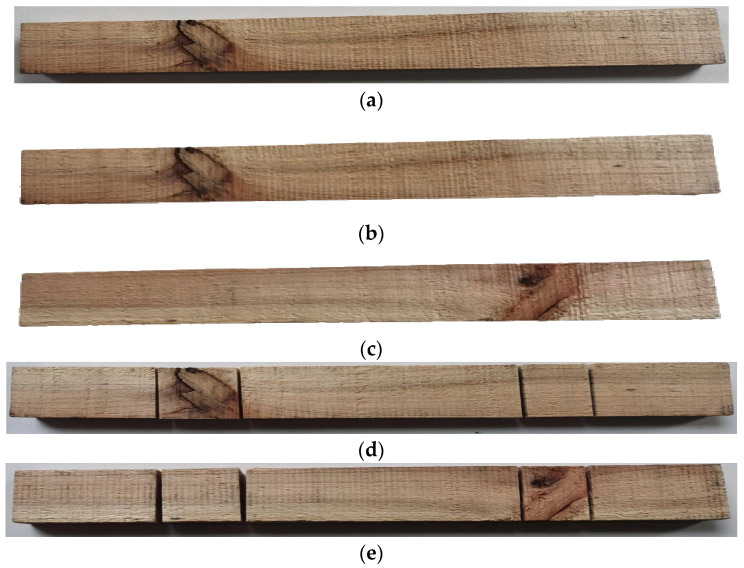
Testing result. (**a**) Solid-sawn lumber; (**b**) top surface image; (**c**) bottom surface image; (**d**) top surface after sawing; and (**e**) bottom surface after sawing.

Figure 12 illustrates solid-sawn lumber undergoing double-sided processing by the device. To elaborate, the solid-sawn lumber, as presented in Figure 12a, was fed into the intelligent defect-oriented processing apparatus. The image acquisition equipment captured the lumber’s upper and lower surface images, as shown in Figure 12b,c. This lumber exhibited two non-coinciding dead knot defects on its upper and lower surfaces. The distance between these two defects exceeded the minimum processing distance, and no waste material issues were encountered. As a result, four sawing operations were required to remove these defects, resulting in the processed solid-sawn lumber depicted in Figure 12d,e.

## 4. Conclusions

The development of deep learning has injected increased vitality into the intelligent selection and processing of solid wood materials. With the continuous advancement of object detection algorithms, more effective means of detecting defects in wood, characterized by its inherently intricate grain patterns and challenging defect identification, have emerged. The image-based apparatus for the defect-oriented processing of solid wood materials stands out due to its simplicity and comprehensive detection capabilities, delivering superior performance.

The application of advanced deep learning technology for wood defect recognition improves wood utilization rates and reduces manual labor. This technology integrates wood processing and manufacturing intelligence into reality, resulting in a 12.3% increase in wood utilization rates compared to fixed-length processing. It also reduces the need for traditional semi-automatic manual scribing. This technology has far-reaching application scenarios. With the advancement of detection technology, other detection methods can be combined with visual detection. A multi-source information fusion method for wood defect location based on confidence was established to improve the defect detection types and accuracy of solid-sawn wood timber. Consequently, this can elevate the processing quality of solid-sawn wood timber and increase the yield rate.

These research results provide an important foundation for the rapid detection and precise removal of defects in solid wood, effectively enhancing its utilization rate. Concurrently, as deep learning algorithms are increasingly applied in practical production, expanding the sample scope in subsequent research is essential. Expanding and enhancing the wood surface image dataset is essential, as most artificial intelligence algorithms are data-driven techniques. On the one hand, a large-scale wood surface image dataset could serve as a source domain for transfer learning applicable to specific products, thereby reducing the data requirements for those products. On the other hand, a more extensive dataset could facilitate the development of lightweight algorithm models tailored for specific solid wood materials, thereby reducing computational costs in practical applications.

## Figures and Tables

**Table 1:** Primary hardware components of the image acquisition platform.

Hardware Name	Hardware Type	Manufacturer information
Illumination	LCOL-300-25	AST-Vision, LCOL-300-25, Shanghai, China
Camera	DALSA LA-GC-02K05B	DALSA, LA-GC-02K05B, Waterloo, ON, Canada
Lens	Nikon 50 mm f/1.8 D	Nikon, Tokyo, Japan

**Table 2:** Defect types and distribution.

Types	Distribution
Crackle	238
Live knot	889
Dead knot	592
Pith	175
Total	1894

**Table 3:** Hardware and software platforms.

Item	Parameter
System	Windows 10 × 64
CPU	Core i7-11700F@2.50 GHz
GPU	NVIDIA GeForce RTX 3080 Ti
Environment configuration	PyCharm + CUDA10.2 + Pytorch1.12.1

**Table 4:** Scores in test set.

Evaluation Index	YOLOv7	YOLOv5	YOLOX
F1	0.93	0.93	0.93
Recall	0.92	0.91	0.92
Precision	0.95	0.96	0.95
mAP@0.5	0.94	0.91	0.92

**Table 5:** Comparative analysis of recall rate of different algorithm models.

Type of Algorithm	Live Knots	Dead Knots	Pith	Cracks
YOLOv7	0.92	0.87	0.94	0.94
YOLOv5	0.90	0.85	0.94	0.94
YOLOX	0.91	0.86	0.96	0.96

**Table 6:** Comparative analysis of precision rates of different algorithm models.

Type of Algorithm	Live Knots	Dead Knots	Pith	Cracks	Mean Average Precision
YOLOv7	0.93	0.89	0.98	0.96	0.94
YOLOv5	0.93	0.80	0.96	0.96	0.91
YOLOX	0.90	0.86	0.96	0.96	0.92

## Data Availability

Data is contained within the article.
